# Spinal cord injury affects the interplay between visual and sensorimotor representations of the body

**DOI:** 10.1038/srep20144

**Published:** 2016-02-04

**Authors:** Silvio Ionta, Michael Villiger, Catherine R Jutzeler, Patrick Freund, Armin Curt, Roger Gassert

**Affiliations:** 1 Rehabilitation Engineering Laboratory, Department of Health Sciences and Technology, ETH Zurich, Zurich, Switzerland; 2 The Laboratory for Investigative Neurophysiology (The LINE), Department of Radiology and Department of Clinical Neurosciences, University Hospital Center (CHUV) and University of Lausanne (UNIL), Lausanne, Switzerland; 3 Spinal Cord Injury Center, Balgrist University Hospital, University of Zurich, Zurich, Switzerland; 4 University College Physiotherapy Thim van der Laan, Landquart, Switzerland; 5 Department of Business Economics, Health and Social Care, University of Applied Sciences and Arts of Southern Switzerland, Landquart/Manno, Switzerland; 6 Department of Brain Repair and Rehabilitation, UCL Institute of Neurology, University College London, London, UK; 7 Wellcome Trust Centre for Neuroimaging, UCL Institute of Neurology, University College London, London, UK; 8 Department of Neurophysics, Max Planck Institute for Human Cognitive, and Brain Sciences, Leipzig, Germany

## Abstract

The brain integrates multiple sensory inputs, including somatosensory and visual inputs, to produce a representation of the body. Spinal cord injury (SCI) interrupts the communication between brain and body and the effects of this deafferentation on body representation are poorly understood. We investigated whether the relative weight of somatosensory and visual frames of reference for body representation is altered in individuals with incomplete or complete SCI (affecting lower limbs’ somatosensation), with respect to controls. To study the influence of afferent somatosensory information on body representation, participants verbally judged the laterality of rotated images of feet, hands, and whole-bodies (mental rotation task) in two different postures (participants’ body parts were hidden from view). We found that (i) complete SCI disrupts the influence of postural changes on the representation of the deafferented body parts (feet, but not hands) and (ii) regardless of posture, whole-body representation progressively deteriorates proportionally to SCI completeness. These results demonstrate that the cortical representation of the body is dynamic, responsive, and adaptable to contingent conditions, in that the role of somatosensation is altered and partially compensated with a change in the relative weight of somatosensory versus visual bodily representations.

Most of us can effortlessly coordinate movements and somatosensations^1^, but worldwide about 180.000 people every year face devastating and in most cases permanent loss of such sensorimotor function as a consequence of traumatic spinal cord injury (SCI)^2^. SCI dramatically affects or even interrupts the communication between the brain and the body and may radically influence body representation^3^—the anatomical reconstruction of the human body in the brain^4^. Whether or not SCI leads to cortical remapping is still an open question^5^ and indeed different studies reported that SCI heavily alters^3^,^6^ or relatively preserves^7^,^8^ the body representation. Such inconsistency might be a consequence of (the underestimation of) the relationship between (impaired) movement execution and (distorted) somatosensory feedback^9^. Indeed, studies in which SCI patients had to execute movements led to contrasting conclusions on affected^10^ versus maintained^11^ body representation. One way to address this problem is to use an investigation tool able to activate central representations without the necessity of movement execution. In this vein, motor imagery (an active mental rehearsal of movements without physical execution^12^) is an ideal cognitive task. Cerebral representations of actions are largely multimodal^13^ and imagined and executed movements show proportional timing (longer movements require longer time to be imagined)^14^ and kinematic configuration (anatomically awkward movements are more difficult to imagine)^15^. In addition, despite specific differences^16^, they engage partially overlapping brain networks^17^,^18^,^19^. A straightforward method to objectively measure motor imagery is the “mental rotation” task, during which images are mentally rotated while reaction times (RTs) and response accuracy are measured. Typically, mental rotation of body parts is sensitive to changes in somatosensory afferent information^20^ in a image-specific fashion^21^. In particular, mental rotation of hand images is assumed to activate somatosensory representations, i.e. the body schema (the representation of touch, proprioception, pain, temperature, interoception, etc)^22^. Conversely, mental rotation of whole-body images seems to rely more strongly on visual representations, i.e. the body image (a pictorial representation of the body, as seen from a third-person perspective)^23^. On this basis, we hypothesized a change in the reliance on somatosensory and visual frames of reference for the construction of body representation based on the available sensory input. To test this hypothesis, we quantified the effects of partial and total peripheral input loss (from the lower limbs, see also Table 1) by comparing the effects of postural changes (hands and feet, straight or crossed) on the mental rotation task (laterality judgment) performed with different bodily images (feet, hands, whole-bodies) after incomplete and complete SCI and in healthy controls.

## Results

**Figure 1:**
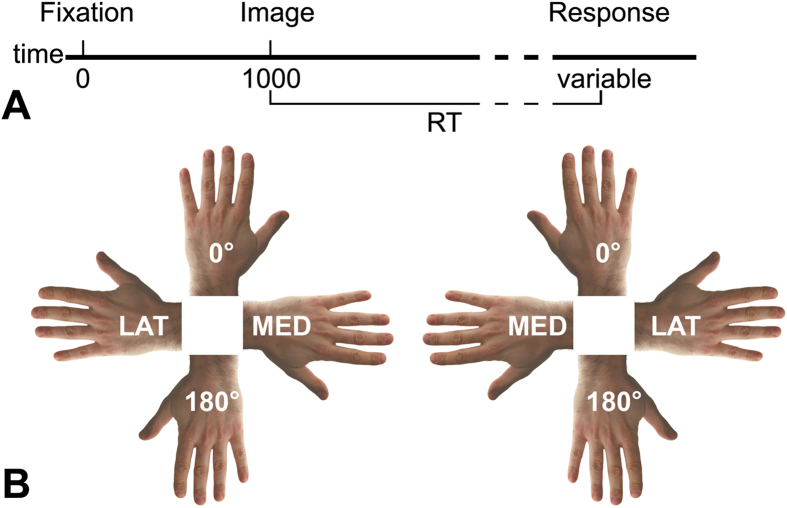
Protocol and stimuli. (**A**) Graphical representation of the task. Images were presented one at a time and remained on the screen until the participant provided the verbal response (left or right). (**B**) Direction of mental rotation. Lateral rotations (LAT) included right-lateralized images at 90° and left-lateralized images at 270°. Medial rotations (MED) comprised right-lateralized images at 270° and left-lateralized images at 90°. Upright and upside-down rotations referred to the fingers’/toes’ orientation (0° and 180°, respectively). For illustration purposes only hand images are shown here. The same applied to foot and body images.

Prior to the experiment, 16 controls, 11 individuals with incomplete SCI, and 11 individuals with complete SCI (Table 1) completed the Vividness of Movement Imagery Questionnaire (VMIQ)^24^ and the Edinburgh Inventory^25^ to assess imagery abilities and handedness, respectively. During the experiment participants verbally judged the laterality (left, right) of rotated (0°, medial, 180°, lateral) images of a foot, hand, and body displayed one at time and from different views (dorsum, planum), while having their own hands and feet in two postures (straight, crossed) (Fig. 1A). Response times (RTs) and accuracy were analyzed according to the factors group (controls, incomplete SCI, complete SCI), posture (straight, crossed), laterality (left, right), view (dorsum, palm/planum), and rotation (0°, medial, 180°, lateral). Lateral rotations comprised right-lateralized stimuli oriented at 90° and left-lateralized stimuli oriented at 270°. Medial rotations comprised right-lateralized stimuli oriented at 270° and left-lateralized stimuli oriented at 90° (Fig. 1B). The correlation between RTs and SCI completeness was also tested. Further information about the methods is reported at the end of this manuscript and in the Supplementary Material.

The VMIQ showed that all individuals with SCI and controls were equally able to perform mental imagery both in first- and third-person perspective (all p > 0.05). No significant differences between the three groups in terms of general imagery abilities were found (all p > 0.05). Similarly, the handedness inventory showed that the three groups were homogeneous in terms of hand dominance, with a strong preference for the right with respect to the left hand (all p < 0.05) and not differing between them in terms of right hand preference (all p > 0.05) and left hand preference (all p > 0.05).

### Mental Rotation of FOOT images

**Figure 2:**
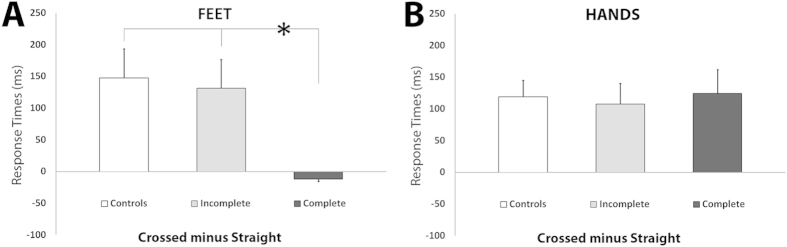
Postural effects on local body representations. (**A**) Posture of Feet posture influences controls’ and incomplete SCI individuals’ mental rotation of feet, but has no effects in the complete SCI group. For illustration purposes, the difference between the response times obtained in the crossed versus straight postural conditions is represented. Positive values represent longer latencies in mentally rotating feet in the (feet) crossed condition with respect to straight. Error bars represent standard errors. (**B**) Posture of Hands posture influences all groups’ mental rotation of hands. For illustration purposes, the difference between the response times obtained in the crossed versus straight postural conditions is represented. Positive values represent longer latencies in mentally rotating hands in the (hands) crossed condition with respect to straight. Error bars represent standard errors. The same color code has been used in Figs 3 and 4 (controls: light grey; incomplete SCI: grey; complete SCI: black).

**Figure 3:**
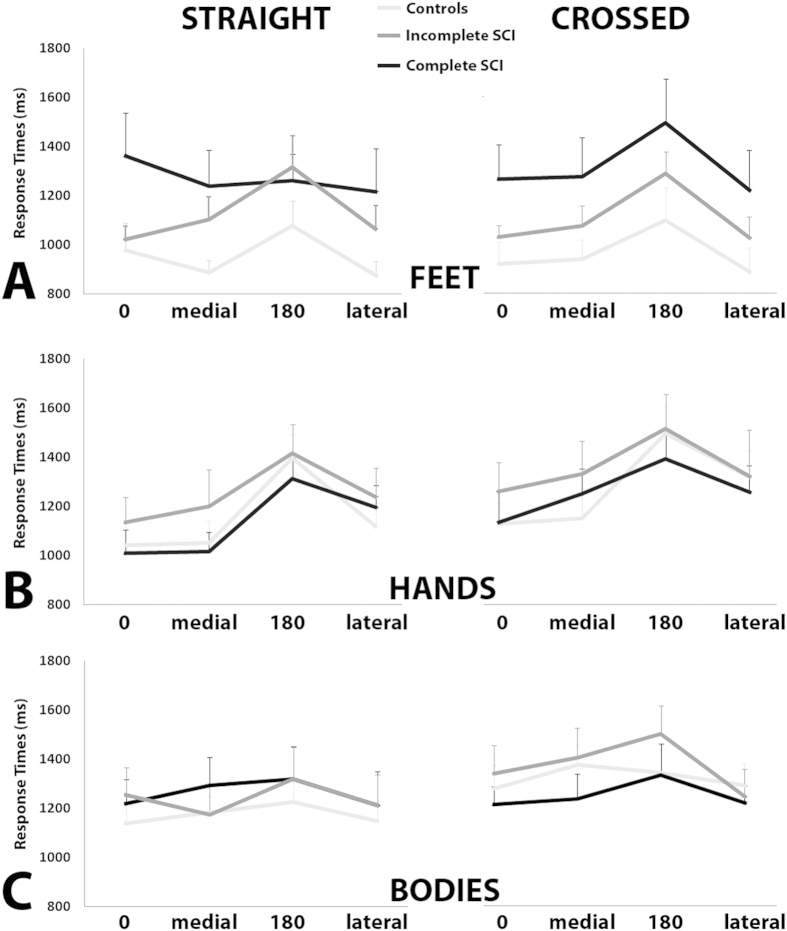
Direction of Rotation. Response times for the mental rotation of feet (**A**), hands (**B**), and bodies (**C**) are plotted as a function of direction of rotation (0°, medial, 180°, lateral) separately for each postural condition (straight, crossed). The same color code has been used in Figs 2 and 4 (controls: light grey; incomplete SCI: grey; complete SCI: black). Error bars represent standard error.

Posture-related effects hinted to a differential weight of somatosensation in the representation of deafferented body parts (feet). The analysis of RTs showed the significant main effect of posture [F(1,35) = 15.7; p = 0.001], accounted for by the overall slower responses in the crossed postural condition (1325 ms) with respect to the straight postural condition (1218ms; p = 0.001). The significant interaction between posture and group [F(2,35) = 4.9; p = 0.013], indicated that only controls and the incomplete SCI group were slower in mentally rotating feet while their own feet were crossed (controls: 1340ms and 1156ms, respectively, p = 0.004; incomplete SCI group: 1378ms and 1227ms respectively, p = 0.031). Conversely, in the complete SCI group the difference in mental rotation task performed in the crossed (1257ms) and straight condition (1272ms) was not significant. In other words, only the complete SCI group failed to show the posture-dependent change in mental rotation of foot images (Fig. 2A). Interestingly, the effect of rotation was very similar between the three groups (all p > 0.05; Fig. 3A). The significant interaction between posture, group, and view [F(2,35) = 4.1530; p = 0.024] indicated that in all groups planum-view images resulted in longer RTs than the dorsum-view images, regardless of posture (all p < 0.05). The accuracy data analysis showed the significant main effect of group [F(2,35) = 4.3; p = 0.023], accounted for by the less accurate performance of the complete SCI group (88%) with respect to healthy controls (96%; p = 0.008). Notably, the difference between controls and incomplete SCI group and between complete and incomplete SCI individuals were not significant (all p > 0.05). The correlation analysis did not show significant interactions between mental rotation task latencies and completeness of SCI. Altogether these results show that, in contrast to controls and incomplete SCI group, complete SCI group’s performance was not modulated by postural changes. Posture-unrelated effects generally confirmed previous findings^21^ and are reported in Supplementary Material.

### Mental Rotation of HAND images

Posture-related effects showed preserved representation of unaffected body parts (hands). RT analysis showed the significant main effect of posture [F(1,35) = 26.5; p = 0.001], accounted for by the slower responses in the crossed (1296ms) with respect to the straight condition (1183ms; p = 0.001). This pattern was significant in each group when tested separately (all p < 0.05) (Fig. 2B). The post-hoc comparisons of the significant interaction between posture, laterality, and rotation [F(3,105) = 4.7; p = 0.004] indicated that in both the straight and crossed postures, the responses for the 180° rotations were slower with respect to all the other rotations (all p < 0.05) (Fig. 3B). The accuracy data analysis showed that the factors group and posture were not significant as main effects or in any interaction (all p > 0.05), indicating that all three groups were equally accurate. The correlation analysis did not show significant interactions between latencies in mental rotation task and completeness of SCI. By highlighting the absence of the group effect both as a main effect or in interaction with other factors, these results suggest that individuals with SCI (both incomplete and complete) and controls processed hand images in a generally equivalent manner, i.e. their performance was sensitive to hand postural changes. Posture-unrelated effects generally confirmed previous findings^20^ and are reported in Supplementary Material.

### Mental Rotation of BODY images

**Figure 4:**
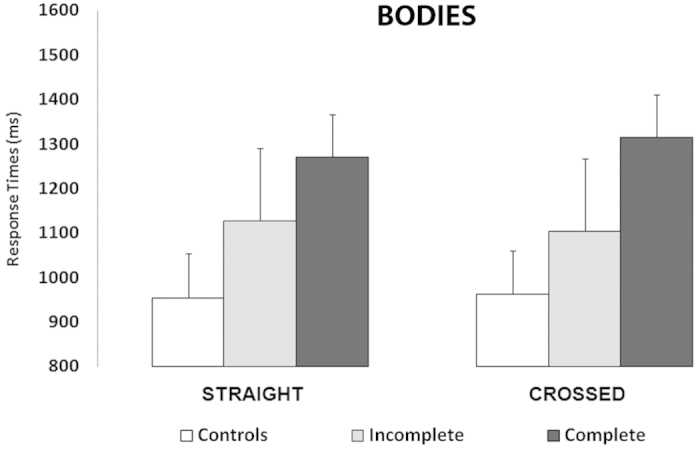
Body rotation. Within each group of participants, response times in the two postural conditions (straight, crossed) were equivalent. Mental rotation of bodies was not influenced by postural changes in neither group. Error bars represent standard errors.

The properties of mental rotation of body images hinted to lesion-dependent adjustments of whole-body representation. Importantly, statistical significance was not reached by the interaction between group and posture either for RTs [F(2,35) = 0.38] or accuracy [F(2,35) = 0.21] (Fig. 4). However, the interaction between group, posture, view, and rotation was significant [F(6,105) = 2.3; p = 0.038]. In particular, regardless of posture, only controls and the incomplete SCI group showed slower responses for the images rotated at 180° with respect to all the other rotations of the same image view (all p < 0.05). Conversely, the complete SCI group showed smaller differences in RTs between the different rotations (Fig. 3C). The accuracy data analysis showed only the significant main effect of rotation [F(3,105) = 7.1; p = 0.001], accounted for by the less accurate performance for the images presented at 180° rotations (83%) with respect to all the other rotations (all p < 0.05), and suggesting that the three groups were equally accurate. This indicates that in either group varying the participants’ posture during the task did not influence the performance in mental rotation. The correlation analysis showed the significant increase of RTs as a function of the completeness of SCI both in the straight (r = 0.34; p < 0.05) and the crossed condition (r = 0.36; p < 0.05), indicating progressively longer latencies in mental rotation of body images for incomplete and complete SCI group, respectively. Posture-unrelated effects generally confirmed previous findings^26^ and are reported in Supplementary Material.

## Discussion

SCI dramatically impairs or even definitively prevents the exchange of afferent and efferent input between the brain and the body^27^,^28^. This intermission can radically affect the way the body is perceived, but the actual state of body representation after SCI is largely unclear. Here, we emphasize that (1) complete paraplegic participants (paralysis and deafferentation of lower limbs but preserved upper limb function) showed selective impairments in the mental representation of feet while the mental representation of hands remained normal, and (2) the whole-body mental representation was proportionally impaired as a function of SCI completeness. These data provide novel insights about the effects of deafferentation on the interplay between somatosensory and visual frames of reference in the construction of body representation.

### Mental rotation of foot images and the relative weight of somatosensation and vision in body representation

The absence of posture-dependent modulation of RTs in the mental rotation of foot images after complete SCI can be interpreted as a sign of a changed relative weight of different frames of reference to represent the body. Thus the weight of somatosensory representations was diminished while the weight of visual representations was augmented. In contrast to a winner-take-all perspective, this interpretation is in line with the idea that we are able to integrate and assign different weights to different sources of sensory information as a function of their reliability^29^. In this vein, in healthy populations the mental rotation task is affected by postural changes, indicating the activation of somatosensory representations. Accordingly, the impact of posture on our data show that controls and the incomplete SCI groups attributed an increased weight to somatosensory aspects of body representation, with respect to visual components. Conversely, the absence of the posture-related effects after complete SCI can be seen as a sign of relative dominance of visuo-spatial (with respect to somatosensory) bodily representations^26^. This interpretation is in line with the conceptual differentiation between the body schema and the body image^30^. The body *schema* refers to the online representation of the somatosensory information about one’s own body^22^. It constitutes the somatosensory and motor component of the body representation^31^ and integrates previous somatosensory and motor experience with current sensory information^32^. Conversely, the body *image* refers to the pictorial component of the body representation^23^, strongly relying on previous visual experience^33^, and combining perceptions and beliefs regarding the appearance of one’s own body^34^. Typically, body schema and body image coherently coexist, but deafferentation may dramatically affect their interplay^35^. Here, based on across- and within-subject comparisons in a statistically powerful sample, we show that, being unable to access somatosensory information of the lower extremities, the complete SCI group’s mental rotation of foot images relied mostly on visuo-spatial transformations (visual imagery) using the body image as the main (but not exclusive) reference frame. Conversely, controls and the incomplete SCI group showed a slowing in mental rotation of foot images in the crossed posture. This suggests that these participants counted mostly on effector-specific (foot) sensorimotor representations and used the body schema as a reference frame. A recent study reported that the sensorimotor brain network is not activated during the mental simulation of movements no longer included in the motor repertoire after SCI^36^, suggesting that mental simulation of movements can be performed also irrespective of the state of the sensorimotor system. Along this line, our data suggest that in presence of complete SCI, there is a change in the relative weight of somatosensory and visual aspects of body representation, possibly due to the unavailability of somatosensory information (from the feet). This lack of information would render less reliable, and therefore overshadow, the body schema in favor of the body image. Accordingly, the specificity of the posture effect (absent for foot images but present for hand images) indicates a fine-tuned recalibration of body representations according to the available somatosensory information and is in line with clinical observations showing impaired mental rotation task as a consequence of sensorimotor deficits^37^,^38^,^39^,^40^.

It might be argued that the heterogeneity of lesion levels in the incomplete SCI group could have affected the correlation between RTs and lesion completeness. However, despite this structural heterogeneity, functionally the large majority of the incomplete SCI group (9 out of 11 individuals) showed homogeneous and only mild somatosensory symptoms at the lower limbs (as measured with the AIS scale; see also Table 1). Of the two remaining participants, one had only unilateral symptoms and one had no sensory symptoms at all. On this basis we considered as very low the possibility that the (absent) correlation was due to heterogeneity.

### Mental rotation of Hand images and the preservation of body specificity

Mental rotation of hand images aimed at testing the specificity of the effects found for the feet^21^. It provided an important control, as hands were mostly unaffected in the involved SCI individuals. As such the interplay between the body schema and body image (see above) should be fully intact and performance should therefore be indistinguishable from controls. This was indeed the case and mental rotation of hand images hinted at the preservation of body specificity: all participants exhibited the posture-dependent modulation of RTs. We interpret this posture-related difference as a sign of preservation of normal movement representations. This interpretation is further supported by the participants’ rotation-susceptible performance as indicated by the significant interaction between posture, laterality, and rotation, showing that the typical performance for mental rotation of hand images^21^ was preserved in both postural conditions. Previous work showed that the influence of biomechanical constraints on the mental rotation task is reflected in the effects of rotation^41^, suggesting the activation of somatosensory processing^42^. Based on the present data, we support that hand representation and related transformations were preserved up to normal standards, further suggesting the importance of an efficient exchange of sensorimotor information between the brain and the body to activate adapted mechanisms for movement planning, simulation, and execution. As for the foot images, it might be argued that the heterogeneity of lesion levels of the incomplete SCI group might have affected their performance in the mental rotation task with hand images. However, in the incomplete SCI group, the AIS scores indicated that the sensory hand function was completely preserved in seven out of eleven individuals, and only mildly affected unilaterally in two individuals and bilaterally in another two individuals (Table 1). In addition, the data showed similar posture-related effects on mental rotation of hand images both in the incomplete SCI and the control groups. Finally, none of the individuals in the complete SCI group showed (functional) somatosensory impairments of the hands (Table 1). For these reasons, it is very likely that the correlation between lesion completeness and RTs for mental rotation of hand images was not significant because the three groups performed similarly, no matter the presence of lesion or not.

### Mental rotation of Body images hints to lesion-dependent reorganization

Among the results on mental rotation of body images, two main points deserve attention. First, the correlation analysis showed that mental rotation of body images slowed down as a function of SCI completeness. In brief, controls had the fastest responses, the incomplete SCI group was at an intermediate level, and the complete SCI group had the longest latencies. Second, posture did not affect any group, in accordance with previous data on the absence of somatosensory influence on mental rotation of body images^26^. Thus, it might be argued that, on the one hand, the correlation analysis showed that the more severe the lesion (reduction of peripheral afferent inflow) the slower the mental rotation task, suggesting the involvement of somatosensory mechanisms. While, on the other hand the absence of postural effects hints at the involvement of visuo-spatial mechanisms. To combine these two apparently contrasting interpretations, it is important to consider the significant interaction between group, posture, view, and rotation. This interaction indicated that in controls and incomplete SCI individuals, the performance was more strongly dependent on the rotation of the images (slower for upside-down images) with respect to complete SCI group. This disadvantage for upside-down bodies (as in our controls and incomplete SCI group) suggests the recruitment of somatosensory mechanisms^43^, while its absence (as in our complete SCI group) indicates the involvement of visuo-spatial processing^42^. Two additional sources of evidence support this differentiation between somatosensory versus visuo-spatial mental processing of bodies. First, visual perception^44^ and mental transformation of full bodies recruit occipito-temporal regions, typically involved in visuo-spatial reasoning^45^. Second, the body schema and body image are integrated and used to create online visuo-motor maps of the body of others^46^. Combining these findings with our data, we suggest that the representation of the whole body is built on information derived from an initial visual inspection and contributes to sending supplementary somatosensory information back to visual encoding modules, interfering with visuo-spatial processing by means of additional input on the current body state^47^. In accordance with this interpretation, previous studies showed that body perception can be altered in conditions of temporary absence of somatosensory input or motor output^48^.

### Modeling sensorimotor impairments in SCI

Here we show the highly selective influence of (limited) somatosensory information (as in paraplegia following SCI) on mental processing of the representation of the disconnected body parts. This influence becomes progressively weaker as a function of (partial or complete) loss of afferent somatosensation. Existing models of sensory-motor integration support the existence of prediction mechanisms able to anticipate the motor^49^ and sensory^50^ consequences of an action^51^. In line with previous evidence suggesting the stronger reliance on visual rather than somatosensory strategies for mental rotation of missing^52^, disconnected^40^ or misrepresented limbs^53^, we propose that when afferent information is not available (as from the feet in complete SCI group), the sensory prediction systems do not receive the required information and become less reliable, triggering a change in the relative weight of somatosensory and visual components of body representation. In other words, given the tight mutual exchange between body schema and body image, if the system processing afferent information is impaired, the brain is able to modify the strategy and adjust the interplay between body schema and body image. In this vein, the brain can attribute more weight to the more reliable frame of reference (as the body image for the complete SCI individuals in the present study) and develop new solutions to interact with the environment, e.g. to guide movements based only on residual visual information after complete sensory deafferentation^54^.

### Insights for basic science and rehabilitation

Body representation after SCI has been considered both as disturbed^55^ and preserved^56^. The origin of such inconsistency might be the underestimation of the association between explicit (attempted) movements and altered sensory processing. Ruling out the confound due to (abnormal) sensory inflow, the mental rotation task solves this methodological issue and allows to study the nature of body representations in SCI in a well controlled manner. The mental rotation task has been used to investigate the properties of body representations in a wide range of neurological disorders affecting cortical regions^38^, subcortical structures^57^, or the peripheral nervous system^37^. These studies showed a correspondence between sensorimotor impairments and the characteristics of the mental rotation task. Very few previous studies used the mental rotation task to study body representation after afferent loss or disconnection^39^,^40^, but their results might be limited by methodological aspects. For instance, Nico *et al*.^39^ focused on amputation and reported increased latencies and decreased accuracy in mental rotation of images representing the dominant amputated hand. However, they presented only hand images (missing a control image) and only total loss of somatosensory information (amputation) was investigated. Conversely, we administered images of different body parts and compared the impact of total and partial somatosensory loss. Fiori *et al*.^40^ focused on SCI and hinted at possible changes in the strategy used to process images of deafferented body parts. However, on the one hand they recorded only accuracy, thus missing important information on implicit measurements as the mental rotation task (e.g. RTs in our study). On the other hand, mostly AIS A patients were included (only two AIS B patients), lacking statistical power for a direct comparison between complete and incomplete SCI. Yet, here we recorded both RTs and accuracy and included statistically powerful samples for both complete and incomplete SCI. Finally and most importantly, neither Nico *et al*.^39^ nor Fiori *et al*.^40^ investigated the effects of postural changes on mental rotation of bodily images, as we did. Based on these methodological advances, here we objectively show how mental rotation task reflects a change in the interplay between different frames of reference for body representations and that this depends on the degree of deafferentation. In line with Nico *et al*.^39^, we confirm an impairment in the representation of deafferented body parts. In addition, our data show that (1) this impairment is specific to the deafferented body part (feet and not hands in the complete SCI group), and (2) there is a gradient in the impairment of the whole body representation as a function of progressively unavailable somatosensory information (correlation between SCI completeness and mental rotation of body images). In accordance with Fiori *et al*.^40^, we support a qualitative change (dominant but not exclusive reliance on somatosensory versus visual representations) in the adopted cognitive strategy (relative weight of body schema versus body image) to mentally represent connected and disconnected body parts. Thus we propose that the mental rotation task (including the comparison of different body parts and different postures) is a reliable tool for the objective assessment of the state of body representations after SCI.

How can the present results be transferred to rehabilitation ? With the main aim of promoting beneficial neural plasticity and restoring brain activity^58^, today’s most used approach for treating SCI is physical training^59^. To foster rehabilitation, traditional mental imagery has shown both positive^60^ and mixed results. This inconsistency might be due to a weak control of the content to mental imagery^61^. Conversely, the mental rotation task provides a more structured method to standardize brain activity and obtain an objective measure (RTs) of cognitive processing. For these reasons the mental rotation tasks might be used in SCI assessment and treatment (i) to better assess the state of the body representation in a screening phase and (ii) to keep the body representations in a “readiness” state and potentially use them to comply with additional interventions for SCI. Indeed, the implementation of mental rotation could be beneficial not only as a complement for physical training, but also for technological solutions such as robotic assistance or neuroprosthetics to alleviate the impact of SCI on daily activities. One of these solutions is the so-called brain-computer interface, a technique able to decode brain activity and translate it into computational commands for external devices^62^. In the context of SCI, a large proportion of non-invasive brain-computer interfaces exploit electroencephalography to decode brain activity^63^, while users are asked to perform uncontrolled mental imagery of movements^64^. In experimental settings this strategy has been proven effective to restore ambulation with a powered wheelchair^65^. However, two main issues render this approach particularly challenging. First, there is no control over the exact content of mental imagery. This aspect might undermine the establishment of stable correspondences between specific neural patterns and particular outputs of the brain-computer interface^66^. Second, the device has to classify different patterns of subject-specific neural activity, but this classification might be problematic due to across-session variability (within-subject) of the relationship between neural activity and decoded motor intent. In combination with adaptive decoders^67^, the implementation of the mental rotation task might be used to reduce both across-session and within-subject variability. In this framework, we propose that the mental rotation task could help to better standardize the content of mental imagery and might render brain activity more reproducible and measurable. Furthermore, including the mental rotation task in rehabilitation procedures for SCI might contrast the deterioration of sensorimotor representations due to disuse, thus maintaining a brain “readiness” e.g. to control movements of prosthetic and orthotic devices.

## Conclusions

SCI interrupts the brain-body interactions but its consequences on body representation are mostly unknown. Here we investigated the influence of SCI on the interplay between somatosensory and visual reference frames in the context of body representation. Our results hint at possible changes in the relative weighting of different source(s) of sensory information on which body representation is grounded. The main outcomes of the present study can be summarized in three main points. First, using the mental rotation task, we provided a quantifiable measure (RTs) of the effects of SCI on body representation. Second, we contrasted local (hands, feet) and global (full-body) representations of the body, specifying the selective effects of SCI on affected versus preserved representations. Third, we directly compared the effects of total and partial somatosensory loss on body representations. On this basis, we further proposed that the present data can be clinically applied, in that mental rotation of bodily images is a direct, non-strenuous, and controlled approach to investigate body representations that could be implemented in standard assessment and rehabilitation protocols.

## Methods

### Procedure

The experimental procedure was approved by the local Ethics Committee of the Canton of Zurich (EK-04/2006) and the study was carried out in accordance with the Declaration of Helsinki. All participants were informed of the study procedures and signed a written informed consent. Prior to the experiment, all the 38 participants (16 controls, 11 individuals with incomplete SCI, and 11 individuals with complete SCI, see also Table 1) completed the Vividness of Movement Imagery Questionnaire (VMIQ^24^) to evaluate the vividness with which they were able to imagine themselves or someone else performing movements, as well as the German version of the Edinburgh Inventory^25^ to assess handedness. During the experimental session participants sat in front of a computer screen positioned 60 cm distant from their eyes. Each trial began with a fixation cross (1000ms). Subsequently, an image appeared on the screen and remained visible until the response was given. Participants verbally judged the laterality (left or right) of each image, as quickly and accurately as possible (panel A of Fig. 1). Response times (RTs) were automatically recorded by a microphone, accuracy was manually entered. Experimental stimuli comprised sets of naturalistic images of feet, hands, and bodies^21^,^26^, oriented in four clockwise orientations (0°, 90°, 180°, 270°). The foot images could be presented from the dorsum or the planum view^21^. The hand images could be presented from the dorsum or the palm view^21^. The body images represented a front-facing standing whole-body with the arms flexed at the level of the elbows and showing either the dorsum or the palm of the hands; one hand of the body image was darker^26^ (participants were asked to identify the laterality of the darker hand). The experimental session comprised six blocks, two blocks for each image type and varied in terms of postural conditions^68^. For the foot images, participants performed the task while having their legs and feet parallel to each other (“straight” condition) and having the legs and feet crossed (“crossed” condition). The posture of the hands did not vary across the two blocks of feet images (always “straight”). For the hand and body images, participants performed the task while keeping each hand on the ipsilateral lap (“straight” condition). As postural manipulation, in the “crossed” condition each hand was placed on the contralateral lap. During all the experimental sessions, hands and feet were always hidden from participants’ view and kept in a palm/planum-down posture. The order of postural conditions and images type was randomized across participants. To familiarize with the task – but avoiding any potential practice bias – before the experiment participants were trained using a set of images different from the ones included in the actual experiment. Posture was not manipulated during the training phase. Additional information is provided as Supplementary Material.

### Subjective data analysis

VMIQ and Handedness scores were analyzed using the two-tailed t-test approach (chi^2^ threshold at p < 0.05). In particular, for the VMIQ first we compared the ability to perform mental imagery in first- and third-person perspective within each group, and then we analyzed the general imagery abilities across groups by averaging the values of the two imagery perspectives within each group and comparing the results between groups. In the same vein, for the handedness inventory we first compared the right- and left-lateralized preferences within each group and then we tested whether the distribution of preferences for one hand was different across groups.

### Objective endpoint measures

RT was defined as the time between the stimulus onset and the onset of the participant’s verbal response. Accuracy was defined as the percentage of correct responses in relation to the total number of trials. Trials with incorrect responses (accuracy) or with RTs falling out of the range between 500ms and 3500ms were excluded from the analyses^21^. Trials excluded because they were incorrect and/or too fast/slow amounted to 8.6% of the total responses. In addition, previous findings showed that mental rotation *towards* the midsagittal plane (medial rotation) has an advantage (shorter latencies) with respect to mental rotation *away* from the midsagittal plane (lateral rotation)^69^. Therefore, we included the factor rotation (lateral, medial) in the following analyses. Thus RTs and accuracy were analyzed by means of six separate 5-way mixed model ANOVA. For each stimulus, the two series of analyses (RTs, accuracy) included group (controls, incomplete SCI, complete SCI), posture (straight, crossed), laterality (left, right), view (dorsum, palm/planum), and rotation (0°, medial, 180°, lateral) as main factors, with repeated measures from the second to the fifth factor. Post-hoc comparisons were carried out using the Newman-Keuls test (threshold at p < 0.05). To investigate the relationship between the performance in the mental rotation task and the completeness of SCI, we performed the Pearson’s correlation analysis between RTs and completeness of lesion for all stimuli and in both postural conditions (threshold at p < 0.05).

## Additional Information

**How to cite this article**: Ionta, S. *et al*. Spinal cord injury affects the interplay between visual and sensorimotor representations of the body. *Sci. Rep.* **6**, 20144; doi: 10.1038/srep20144 (2016).

## Supplementary Material

Supplementary Information

## Figures and Tables

**Table 1:** Sample classification.

**#**	**Diagnosis**	**Level**	**Lesion**	**AIS**	**Sensory Impairment**		**Years**
					**Upper limbs**	**Lower limbs**	
1	Para	T7	Com	A		***	16
2	Para	T7	Com	A		***	24
3	Para	L1	Com	A		***	13
4	Para	L2	Com	A		***	17
5	Para	T12	Com	A		***	11
6	Para	T3/T4	Com	A		***	7
7	Para	T11	Com	A		***	19
8	Para	L1	Com	A		***	18
9	Para	T2	Com	A		***	7
10	Tetra	T5	Com	A		***	22
11	Tetra	C7	Com	A		***	13
12	Para	T7	Inc	B		*	26
13	Para	L3	Inc	B		*	12
14	Para	L2	Inc	B		*	6
15	Para	T12	Inc	D		*	2
16	Para	L1	Inc	D		*	19
17	Para	L3	Inc	D		*	5
18	Para	L4	Inc	D		*	2
19	Tetra	C6/C7	Inc	B	*	*	6
20	Tetra	C7	Inc	D	* (right)		11
21	Tetra	C2	Inc	D	*	*	12
22	Tetra	C4	Inc	D	* (right)	* (right)	10

Clinical variables of the involved individuals with SCI, including: the general classification as paraplegics and tetraplegics (Diagnosis; Para = paraplegia, Tetra = tetraplegia); the lesioned segment of the spinal cord (“Level”; C = cervical, T = thoracic, L = lumbar); the completeness of the SCI (Lesion; Com = complete, Inc = incomplete); the general score obtained at the AIS questionnaire (AIS); the somatosensory impairments (Sensory Impairment) in the upper and/or lower limbs (*** = severe, * = mild) evaluated with the AIS scale^65^; and the time since the SCI lesion at the moment of the experiment (Years).
