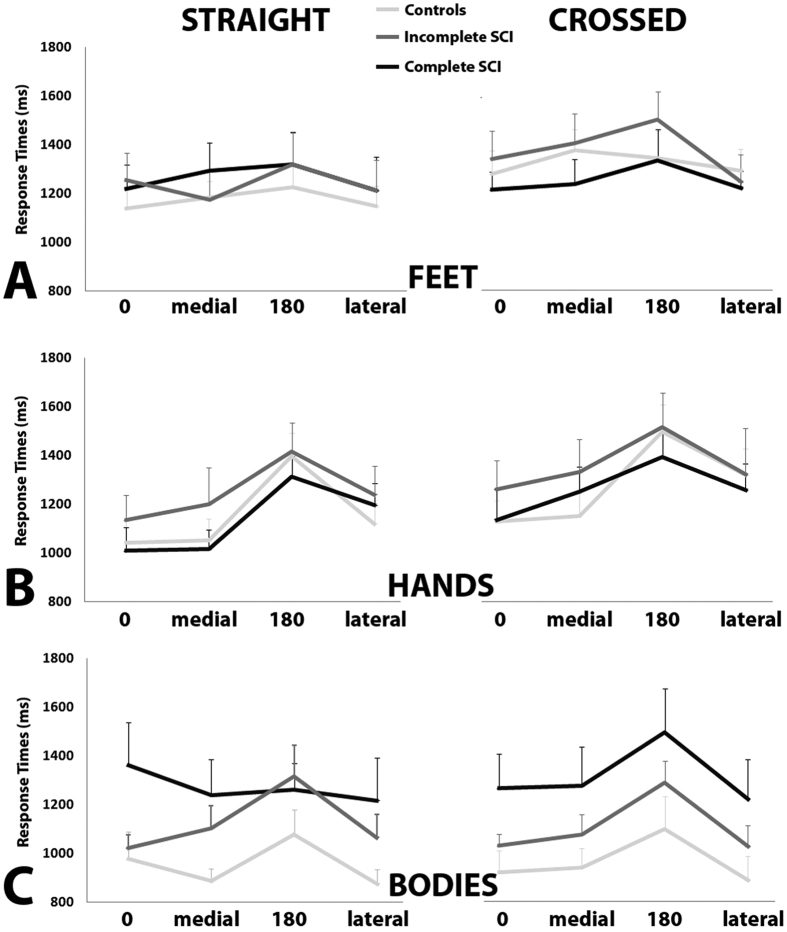# Corrigendum: Spinal cord injury affects the interplay between visual and sensorimotor representations of the body

**DOI:** 10.1038/srep24693

**Published:** 2016-05-20

**Authors:** Silvio Ionta, Michael Villiger, Catherine R. Jutzeler, Patrick Freund, Armin Curt, Roger Gassert

Scientific Reports 6: Article number: 20144; 10.1038/srep20144 published online: 02042016; updated: 05202016.

In this Article, Figure 3 is incorrect. The correct Figure 3 appears below as Fig. 1.

## Figures and Tables

**Figure 1:**