# Supplementary material for: Spinal cord injury affects the interplay between visual and sensorimotor representations of the body
Source: Sci Rep. 2016 Feb 4;6:20144. doi: 10.1038/srep20144 (PMC4740737; doi:10.1038/srep20144)
Supplement: Supplementary Information [file srep20144-s1.docx]

**Spinal cord injury affects the interplay between visual and sensorimotor representations of the body**

Silvio Ionta^1,2,§,*^, Michael Villiger^3,4,5,§^, Catherine Jutzeler^3^, Patrick Freund^3^, Armin Curt^3,¥^, Roger Gassert^1,¥^

1. Rehabilitation Engineering Laboratory, Department of Health Sciences and Technology, ETH Zürich, Zurich, Switzerland.
2. The Laboratory for Investigative Neurophysiology (The LINE), Department of Radiology and Department of Clinical Neurosciences, University Hospital Center (CHUV) and University of Lausanne (UNIL), Lausanne, Switzerland.
3. Spinal Cord Injury Center, Balgrist University Hospital, University of Zurich, Zurich, Switzerland.
4. University College Physiotherapy Thim van der Laan, Landquart, Switzerland.
5. Department of Business Economics, Health and Social Care, University of Applied Sciences and Arts of Southern Switzerland, Landquart/Manno, Switzerland.

* Corresponding Author

§ These authors equally contributed

^¥^ Co-senior authors

All correspondence should be addressed to:

**Silvio Ionta**

CHUV - Nestle Hospital - Avenue Pierre Decker 5

1011 Lausanne, Switzerland

voice: +41213148085

email: [ionta.silvio@gmail.com](mailto:ionta.silvio@gmail.com)

**SUPPLEMENTARY MATERIAL**

**Methods**

*Procedure*

Between the two views of each image the overall configuration was very similar in terms of visual features such as shape, size, and luminosity. The left-lateralized images were mirror images of the right-lateralized ones. All images covered a visual angle comprised between 11° and 13°. Image presentation was controlled by the E-Prime 2 Pro software (Psychology Software Tools Inc., Pittsburgh USA). Each block contained 48 images of the same type (foot, hand, or body) randomly presented in one of four orientations. Within each block, images varied in terms of laterality (left, right), view (dorsum, palm), and orientation (0°, 90°, 180°, 270°). Each image (left and right), in each view, and rotated in each orientation, was presented 3 times within each block (with the same combination of laterality, view, and orientation never repeated twice in sequence).

**Results**

*Mental rotation of FEET*

Response Times - The laterality [F(1,35)=8.6; p=0.006], view [F(1,35)=151.7; p=0.001], and rotation [F(3,105)=4.2; p=0.008], as well as the 2-way interactions between view and rotation [F(3,105)=37.6; p=0.001] and between laterality and view [F(1, 35)=4.7; p=0.48] were significant. The main effect of laterality was accounted for by the slower responses for the left-lateralized stimuli (1306ms) with respect to the right-lateralized ones (1237ms; p=0.005). The significant view effect was explained by the slower responses for the planum-view (1463ms) than the dorsum-view stimuli (1081ms; p=0.001). The post-hoc comparisons of the main effect of rotation showed that the slowest responses were provided for the 180° rotations (1345ms) with respect to all the other rotations (all p<0.05), which did not significantly differ among each other (all p>0.05). The post-hoc comparisons of the interaction view by rotation showed that the stimulus view completely reversed participants’ responses (all p<0.05). The post-hoc comparison of the interaction between laterality and view indicated that participants’ responses to planum-view stimuli were slower for both right- and left-lateralized stimuli (all p<0.05). Altogether these data support and further extend previous data on mental processing of images of feet, showing an equivalent modulation of mental processing of feet as a function of the stimulus view and rotation regardless the presence of SCI.

Accuracy - The main effect of view was significant [F(1,35)=14.9; p<0.001], showing the less accurate responses for the stimuli presented from the planum-view (87%) with respect to the dorsum-view (95%; p=0.001). In addition, we also found the significant 2-way interactions between view and rotation [F(3,105)=5.7; p=0.001] and 3-way interaction between group, view, and rotation [F(6,105)=3.2; p=0.005]. Post-hoc comparisons of the view by rotation interaction showed that within each view the 180° rotations were significantly different from the others (all p<0.05). The post-hoc comparisons of the group by view by rotation showed that only in the complete SCI group the responses to dorsum-view stimuli were more accurate than planum-view for 0° (p=0.001), medial (p=0.001) and lateral rotations (p=0.002). No view-related significant difference was found for the 180° rotations (all p>0.05).

*Mental rotation of HANDS*

Response Times - The main effect of rotation [F(3,105)=34.3; p<0.001] and the 2-way interaction between view and rotation [F(3,105)=12.1; p<0.001] were significant. The main effect of rotation was accounted for by the slowest responses for the 180° rotations (1424ms) with respect to all the other rotations (all p<0.05) as well as by the fastest responses for the 0° rotations (1120ms) with respect to all the other rotations (all p<0.05). The post-hoc comparisons of the view by rotation interaction showed that for the dorsum-view stimuli the RTs were strongly affected by the rotation, with all the responses significantly different between rotations [0° (1005ms), medial (1186ms), 180° (1492ms) lateral (1171ms); all p<0.05)], except medial versus lateral rotations (p>0.05). Conversely, the RTs for the palm-view stimuli were weakly influenced by rotation, the 180° rotation being the slowest (1356ms) with respect to all the others (all p<0.05). Confirming and extending previous data on healthy subjects these results show that, in the presence of residual somatosensory inflow, the impact of stimulus view on mental spatial transformation of hands is equivalent to normal conditions even after SCI.

Accuracy - The main effects of view [F(1,35)=6.1; p<0.021] and rotation [F(3,105)=20.2; p<0.001] were significant. The main effect of view was accounted for by the more accurate performance for stimuli presented in the palm-view (89%) with respect to the dorsum-view (85%; p<0.023). The main effect of rotation was explained by the least accurate performance for the 180° rotations (77%) with respect to all the other rotations (all p<0.05). These results show that all three groups were equally accurate.

*Mental rotation of BODIES*

Response Times - The main effects of laterality [F(1,35)=15.0; p<0.001], view [F(1,35)=5.5; p<0.022], and rotation [F(3,105)=11.5; p<0.001] were significant. The main effect of laterality was accounted for by the faster responses for the right-lateralized stimuli (1084ms) with respect to the left-lateralized ones (1159ms; p<0.05). The main effect of view was explained by the faster responses for the dorsum-view stimuli (1104ms) with respect to the palm-view ones (1139ms; p<0.021). The main effect of rotation was accounted for by the slowest responses for the 180° rotations (1256ms) with respect to all the other rotations (all p<0.05). These results generally confirm previous data indicating facilitation for right-lateralized and dorsum-view body stimuli, as well as increased RTs for upside-down stimuli.

Accuracy - Only the main effect of rotation [F(3,105)=7.1; p<0.001] was significant, accounted for by the less accurate performance for the stimuli presented at 180° rotations (83%) with respect to all the other rotations (all p<0.05).
